# Is Behavioural Therapy a New Treatment Option for Task-Specific Dystonia in Athletes? A Case Series

**DOI:** 10.5334/tohm.737

**Published:** 2023-05-08

**Authors:** Marleen Ieke Tibben, Erik van Wensen, Beorn Nijenhuis, Johannes Zwerver

**Affiliations:** 1 HSK Expertise Center Functional Movement Disorders, Woerden, The Netherlands; 2 Gelre Hospital & Sports Dystonia Centre, Apeldoorn, The Netherlands; 3 University of Groningen, Groningen, The Netherlands; 4 Center for Human Movement Sciences, University Medical Center Groningen, University of Groningen and Department Sports and Exercise Medicine, Sports Valley, Gelderse Vallei Hospital, Ede, The Netherlands

**Keywords:** Task-specific dystonia, athletes, behavioural therapy, sports, golf, yips, runner’s dystonia

## Abstract

**Background:**

Task-specific dystonia is a movement disorder of the central nervous system characterized by focal involuntary spasms and muscle contractions, which can negatively affect performance of a specific task. It can affect a wide range of fine motor skills, also in athletes. Current management of task-specific dystonia includes mainly prescribing drugs, exercise therapy or botulinum injections to the affected muscles. Psychological interventions for athletes suffering from task-specific dystonia have not been described extensively so far.

**Methods:**

We present a case-series of 4 different advanced skill-level athletes with suspected task-specific dystonia, which had a major impact on their performance. They all received treatment consisting of a combination of standardized behavioural therapy and relaxation techniques in the form of hypnosis in a total of 8 sessions in a 16-week time period.

**Results:**

After treatment, all athletes returned to their original high level of sport performance without further symptoms of their suspected task-specific dystonia.

**Discussion:**

Behavioural therapy in combination with a relaxation technique seems to be a safe and promising treatment for athletes with suspected task-specific dystonia. Further studies in a larger, preferably randomized controlled trial, are warranted to evaluate if this treatment strategy is effective in athletes with suspected task-specific dystonia.

## Introduction

Task-specific dystonia (TSD) has been accepted as a neurological movement disorder since the end of the 20^th^ century thanks to the contributions of David Marsden [1]. TSD is a form of dystonia which has been characterized as a type of isolated focal dystonia [2] and is characterized by focal involuntary spasms and muscle contractions in a part of the body that cause unintended and abnormal movements and/or postures during the performance of a specific highly-skilled motor task [3].

The most prevalent feature is a high degree of repetitive muscularly over-active movement by a body part performing a highly trained skill, such as hands, arms, feet or mouth. TSD affects tasks that require accuracy and a great deal of repetitive practice to be mastered [4]. The most well-known example is writer’s cramp [1,5]. TSD can also occur in various other professions, including musicians, hairdressers and typists [6]. Among athletes, several types of possible TSD have been described in professional [7] and lay literature, all with sports related characteristic terminology like runner’s dystonia and skater’s cramp [8]. It is primarily a clinical diagnosis, since there are no proven diagnostic tests for this condition, although co-contraction of antagonistic muscle-pairs can be a feature [9]. Other noted features: it is more common in men and it usually affects the head or upper extremity [10]. In some individuals, the onset of TSD is precipitated by a triggering event [11]. For musicians, such events include a change in playing technique, injury or a new instrument with a slightly different dimension [12]. The consequences of developing the disorder is considerable. Performances where a high degree of motor control are essential such as in sports or music will often experience decrements in ability so severe they might even be forced to end their careers.

Currently, there is continued debate on this issue of etiology, especially in forms of TSD in sports [9]. Research on musicians suggest that both genetics, behavior, and personality traits are important [13]. Behavior, including repetitive over-practicing of a motor task as well as personality traits, such as perfectionism and anxiety, predispose people to TSD [14,15].

Various treatments for TSD have been reported [16,17]. Prescribing drugs such as benzodiazepines, trihexiphenidyl, beta-blockers or injections with botulinum-toxin are commonly used. Botulinum neurotoxin (BoNT) type A has been the mainstay of treatment for TSD [18]. Many studies have demonstrated treatment benefits of BoNT in TSD, but it is difficult to reduce dystonic symptoms without inducing concurrent residual weakness resulting in loss of motor function [12,19,20,21,22]. Occasionally Deep Brain surgical procedures such as Ventro-Oral Thalamotomy and Globus Pallidum DBS are used with positive results in several case-reports [23,24].

Since current treatment options are not always successful, we propose new strategies should be explored. Thus far, the effect of psychological interventions in the form of behavioral therapy in TSD has not been investigated extensively. However there are examples of succesfull treatments that appear to suggest that a psychological intervention might be effective [25]. In our study we attempted to intervene on a psychological level, using behavioral therapy and a relaxation technique by means of hypnosis, on a group of athletes with a suspected TSD, as our major research question was wether this intervention could improve TSD and especially if it would have a positive effect on athletes with TSD.

So the aim of this case report is to investigate if behavioural therapy in combination with a relaxation technique is a promising treatment for athletes with suspected TSD.

## Methods

### Participants

Between February 2018 and December 2019 four different advanced skill-level athletes with suspected task-specific dystonia, were included in the initial study sample. These four participants contacted a neurologist with special interest in task-specific dystonia in sports. Neurological examination took place at Gelre Hospitals. Patients were eligible for the treatment program if the neurologist diagnosed the patient with a possible TSD with an otherwise normal routine neurological examination. The neurologist referred the athletes to HSK Center of Expertise for Functional Movement Disorders, a Dutch Healthcare institution specialized in the treatment of patients with movement disorders. A specialized psychologist with ample expertise in movement disorders performed the intake and treatment.

During the intake, the short version of the Schedules for Clinial Assessment in Neuropsychiatry (mini-SCAN) questionnaire was administered [26]. Based on the mini-SCAN, psychological comorbidity was excluded. Exclusion criteria defined as: an insufficient understanding of the Dutch language, suicidal tendencies, comorbidity with a psychotic disorder, substance use disorder and/or a severe depression at the time of the intake.

This study was conducted in accordance with applicable law and reviewed by an accredited research ethics committee. As no variables were manipulated, the provided treatment was considered ‘usual care’ and patient investment was minimal. Patients gave informed consent at the intake for presenting their medical history and for the videos.

#### Treatment intervention

The therapy-course consisted of 8 sessions in 16 weeks and a follow up. The treatment included a combination of behavioral therapy (BT) and relaxation techniques in the form of hypnosis. The duration of a session was 90 minutes. Components of treatment were psycho-education, shaping, hypnosis and relapse prevention. In each session a new hypnosis exercise was introduced and practiced. The treatment was a tailored combination of interventions, slightly different for each athlete.

Upon the first therapy session following the intake, hypnotic principles were explained to the athletes. Consequently an introductory hypnosis exercise was performed to introduce athletes to the hypnotic trance. During the subsequent sessions an incompatible response specific to the athlete’s symptoms was taught and induced in hypnosis. The aim of the exercises was to relax hands, upper arms, shoulders, feet or legs depending on the location of the specific TSD. After 4 or 5 sessions, athletes learned to evoke the incompatible response without any formal induction. The exercises were recorded so the athletes could practice them at home. They were recommended to practice 5 to 10 times a day. Subsequently, the exercises were made more difficult, and the athletes learned to relax under more stressful circumstances, such as during competitions. A relapse prevention plan was created with the athlete what to do if TSD occurred.

## Results

### Case 1: A golfer with the Yips

A competitive male golfer (age 40) was unable to play at his normal level, experiencing reduced control over his left arm while playing, as well as increased tension in his entire body while putting. The involuntary movements first arose during a golf clinic and continued for 13 years. Practicing more frequently increased the occurrence and speed of onset of the involuntary movements. Due to these movements he had to stop playing at competitive level. This also had an impact on his physical and psychological condition. He used propranolol at a low dosage of 10 or 20 mg before competition, but this was ineffective.

After a clinical examination no neurological abnormalities were found. During a video-recording session, dystonia was observed in the left arm while performing a putt with the left hand (see video 1). The diagnosis of TSD in golf, the “Yips”, was made. No comorbid psychiatric conditions were found during intake.

**Video 1:** Before treatment: Male golfer, showing reduced control of his left arm as he is putting with only his left arm, putting from right to left. Phenomenology: Decreased motor control appears to be due to simultaneously increased tension of different antagonistic muscles of the left arm. This pattern of co-activation suggests a task-specific dystonia in his left arm while putting.

During the treatment sessions several behavioural and relaxation techniques in the form of hypnosis, treatment strategies were implemented, aiming to relax his hands, upper arms, shoulders and back. Playing golf was prohibited until after the 6^th^ session. Following the 7^th^ session, he reported having played 2 rounds of golf, free from any yips-symptoms; furthermore, his game had improved compared to recent years. Importantly, also enjoyment for the game had returned. Five years after completion of treatment, the golfer was almost free of symptoms (see video 2). Occasionally, during a match, when he was under pressure, the yips still occurred. At these moments he was able to control the symptoms by applying the above-mentioned relaxation techniques.

**Video 2:** After treatment: The same golfer showing normal motor control in his left arm while putting with only his left arm. Phenomenology: There is no movement disorder visible.

### Case 2: A billiard player with “Cueïtis”

A male billiards player (age 53) suffered from an involuntary movement in his right arm, exclusively while playing billiards. He had played billiards at a competitive level for 39 years. Symptoms began after he had taken lessons from a professional billiards player and exacerbated during competition. He reported a loss of control over the cue, as he was no longer able to move the cue smoothly through the range of motion of an average stroke. He had been suffering from this involuntary movements for the last 5 years. He tried propranolol for a month, in a dosage of 30mg, 1 hour before the game, without effect.

The neurological examination was normal. A video recording showed striking the billiard ball without any pre-stroke movements, because of his complete physical ‘blocking’ (see video 3). The diagnose of TSD in billiards, “Cueïits”, was made and BT sessions were started.

**Video 3:** Before treatment: Male billiard-player, demonstrating the inability to move the cue smoothly, back and forth, through the range of motion before hitting the ball. Phenomenology: There is no movement disorder visible.

No comorbid psychiatric conditions were found during intake. During treatment the billiards player was encouraged to practice engendering a ‘light and flexible’ feeling of his right arm, thereby reducing and possibly limiting the involuntary muscle contractions.

After 3 sessions the billiard player reported performance improvements, not only during non-competitive matches, but also in competition, resulting in a league-win (after 14 lost matches). Upon his last session he reported that involuntary muscle contractions still occurred in 10% of all games, but he had reached a stable level of acceptance regarding his current level of play. At 5-years follow-up, he indicated he still had benefit from the acquired skills and that he could hit the ball with a complete normal stroke (see video 4). He reported that once in a while there were moments of blocking while punching with the cue. By applying his learned psychological techniques, the involuntary contractions disappeared.

**Video 4:** After treatment: Male billiard-player, demonstrating the normal pre-shot movements as he moves the cue smoothly, back and forth, through the range of motion before hitting the ball. Phenomenology: There is no movement disorder visible, but the difference with video 3 is striking.

### Case 3: A runner with “Runner’s Dystonia”

A female runner (age 49) suffered from problems controlling her legs during running. Symptom onset usually occurred after approximately 2 kilometers of running. She was no longer able to lift her legs properly and started dragging. Initially, she was able to ‘push through’ with disappearance of symptoms later on in the run, but eventually symptoms persisted throughout the run. Problems with the coordination had started 7 years ago. The involuntary movements started during a training, where she was asked to raise her knees higher while running. The symptoms had increased in recent years. No abnormalities were found during the neurological examination and the diagnosis “Runner’s Dystonia” was made. BT sessions were started.

During intake no comorbid psychiatric conditions were found. At treatment onset, the runner got the advice to temporarily stop running completely. She started doing a series of exercise to encourage a feeling of smooth, loose, and firm hips and legs. The runner then practiced to evoke this feeling while standing, and later on during jogging. Whenever she noticed the beginning of the coordination problems, she paused to recall the practiced feeling and continued jogging. During the sixth session she was able to run a total of 5 kilometers (interval running and walking). At 3 months follow up the runner was able to run 6 kilometers without symptoms. By applying the aforementioned technique, she was able to gradually increase her symptom-free running distance. Unfortunately we do not have video-recordings of her running pattern before and after treatment.

### Case 4: A skater with “Skater’s cramp”

A female speed skater (age 19) suffered from a regular and patterned jerking movement in her right foot while skating. The jerking movement consisted of a sudden unconscious exorotation of her foot, and this always occurred at exactly the moment the skater’s skate was being placed back on the ice after a completed stroke. The skater had begun skating at the age of 6. Unsuccessful attempts to alleviate the problem were done, including taping her right foot, and using her old skating equipment. The involuntary movements had persisted for almost a year before treatment began. During neurological examination using video recording of her speed skating, an involuntary movement in her right foot was documented (see video 5). The diagnosis TSD in skating; “Skater’s cramp”, was made and BT sessions were started. During the intake no comorbid psychiatric conditions were found.

**Video 5:** Before treatment: Female speed skater, showing a regular and patterned jerking movement in her right foot as she is skating in a straight line. The jerking movement consists of a sudden unconscious eversion of her foot that always occurrs at exactly the moment the skater’s skate was being placed back on the ice. Phenomenology: Decreased control appears to be due to repetitive jerking by simultaneously increased tension in different antagonistic muscles of the right leg followed by eversion of the right foot. This pattern of co-activation suggests a task-specific dystonia in her right foot while skating. Differential diagnoses: a task-specific myoclonus or functional movement disorder.

Exercises were provided to relax her lower legs completely as she experienced the cramping. In addition, because of the deviation of her right foot on the ice during skating, she was encouraged to imagine that her right foot was made of steel, and in her mind she placed this foot in the correct way on the ice. After 5 sessions, an attempt was made to apply the techniques in practice. Results seemed promising and after 8 sessions she was able to skate without the cramping movement.

During high intensity skating (sprinting), there were still minor indications of a cramping movement, but overall, she reported vast improvements across the entire spectrum of her skating (see video 6). At 4 months follow-up, skating had even further improved, with no marked indications of a skater’s cramp and several new personal records. Five years after treatment she is still symptom free from the TSD.

**Video 6:** After treatment: Same skater showing normal skating pattern in both legs while skating. Phenomenology: There is no movement disorder visible.

## Discussion

In this case series of four athletes from different sports, diagnosed with a probable TSD, a combination of behavioral therapy and a relaxation technique reduced symptoms, improved sport-specific function and restored a much better control over their skilled movements.

The involuntary spasms and muscle contractions that occurred prior to treatment were task-specific and were not accompanied by any neurological abnormalities, making alternative neurological reasons for the disorder unlikely. After treatment marked improvements were observed in symptoms and a much better control over their practiced movements. Post-treatment, even at more than 4-years follow-up, participants reported no or minimal problems with their diagnosed TSD, and no side effects or worsening of the condition were reported.

TSD is a movement disorder that is not fully understood. Limitations of neural circuitry supporting skill expertise are likely to be an important contributory factor in the development of TSD [27], particularly in professional musicians and athletes. Part of the problem has been suggested to be linked to errors in highly complex task-specific motor programs. In frequent rehearsed tasks stereotyped sequences begin to dominate the movement repertoire. As a result conferred flexibility for related tasks could become redundant [27]. Longer motor programs may develop from over training and it is suggested that these might be more susceptible to developping errors that lead to TSD. If dysfunctional or dystonic movements are repeatedly practiced they will become encoded in a similar manner to any other sequence of movements [27].

Behavior therapy is a technique for changing behavior in a constructive way, by means of applying new learned strategies. In our athlethes with a TSD it was the intention to interrupt a disturbed motor program producing an involuntary spasm, by applying desired behaviour, supported by relaxation techniques, in this case by applying hypnosis because hypnosis makes it possible to relax the muscles that are involved in different forms of TSD.

An important prerequisite for successful treatment seemed to be the motivation of the athletes. The treatment is time intensive and therefore it is important they are motivated to practice extensively with the techniques provided. Since they were used to intensive training, this was not a problem for the athletes in this series. We expect this will not be a problem for most (elite) athletes, although it is an important point of consideration for recreational athletes.

This is, to our knowledge, the first case series which describes promising results for behavioral therapy and relaxation techniques in sports-specific TSD; however, these results should be interpreted with some caution. We describe only a very small group of four athletes with four possible sport-specific TSD’s. Secondly, TSD remains a clinically diagnosed condition, for which there is no consensus on a validated set of diagnostic criteria yet [28]. Furthermore, no specific sports related, validated TSD questionnaires are available. So to evaluate the possible effectiveness of behavioural therapy and relaxation techniques, more extensive prospective and controlled studies, using well defined diagnostic criteria and validated outcome measures among larger groups of athletes with TSD are needed. Ultimately this will help to find the best treatment strategies for athletes who suffer from a sport-specific TSD.

We conclude that task-specific dystonia is a heterogenous, but still very mysterious movement disorder which also seems to affect athletes of various sports [8], thereby worsening their performance, as well as their ‘joy of play’. Behavioural therapy in combination with a relaxation technique seems to be a safe and promising treatment for athletes with a suspected task-specific dystonia. Further studies in a larger cohort, preferably in a randomized control trial, are warranted to evaluate if this treatment strategy is also effective in other athletes diagnosed with task-specific dystonia.
